# sTim-3 alleviates liver injury via regulation of the immunity microenvironment and autophagy

**DOI:** 10.1038/s41420-020-00299-7

**Published:** 2020-07-22

**Authors:** Ying Yang, Gaoxiang Ying, Fengtian Wu, Zhi Chen

**Affiliations:** 1 State Key Laboratory for Diagnosis and Treatment of Infectious Diseases, The First Affiliated Hospital, College of Medicine, Zhejiang University, 310003 Hangzhou, China; 2 National Clinical Research Center for Infectious Diseases, The First Affiliated Hospital, College of Medicine, Zhejiang University, 310003 Hangzhou, China; 3 Collaborative Innovation Center for Diagnosis and Treatment of Infectious Diseases, The First Affiliated Hospital, College of Medicine, Zhejiang University, 310003 Hangzhou, China

**Keywords:** Preclinical research, Translational research

## Abstract

Liver failure (LF) is a monocyte/macrophage-mediated liver injury that has been associated with inflammatory mediators. However, the mechanism through which monocytes/macrophages regulate LF has not been fully elucidated. In this study, we investigated the role of soluble T-cell immunoglobulin domain and mucin domain-containing molecule-3 (sTim-3) in inhibition of release of inflammatory mediators. We further assess this role in protection against D-galactosamine (D-GalN)/lipopolysaccharide (LPS)-induced acute liver failure (ALF), via monocyte/macrophage regulation and autophagy induction in mice. Our findings indicate significantly higher plasma sTim-3 in acute-on-chronic liver failure (ACLF) group relative to other groups, with this trend associated with disease progression. Furthermore, infiltrated recombinant sTim-3 inhibited release of various inflammatory mediators, including cytokines and human high-mobility group box-1 (HMGB1), potentially via autophagy induction. Furthermore, H&E staining and the low levels of alanine aminotransferase (ALT) and aspartate aminotransferase (AST) in ALF mice, supported that recombinant sTim-3 effectively alleviated liver injury. Moreover, sTim-3 induced changes in monocyte/macrophage population in mice’s liver or blood, which consequently caused a reduction in proinflammatory CD11b^hi^F4/80^lo^ monocyte-derived macrophages and Ly-6C(+)CD11b(+) monocytes. Conversely, sTim-3 increased autophagy levels of hepatic CD11b(+) monocyte-derived macrophages and decreased apoptosis rate of CD11b (+) monocytes in the blood. Collectively, our findings demonstrated that sTim-3 alleviated inflammatory response and liver injury by promoting autophagy and regulating monocyte/macrophage function. This indicates its potential for future development of novel therapeutic strategies against LF.

## Introduction

Acute-on-chronic liver failure (ACLF) is one of clinical manifestations of liver failure (LF) with acute deterioration of cirrhosis, and results from a precipitating event, and subsequently leads to multiple organ failure^1^. Approximately one third of all ACLF cases are linked to bacterial infection, whereas about 20% of acute liver failure (ALF) result from drugs^2^. Previous studies have described the use of D-galactosamine (D-GalN) and lipopolysaccharide (LPS) in establishment of experimental ALF models^3^. In addition, LPS is one of the bacterial pathogen-associated molecular patterns (PAMP), which can also exacerbate ACLF by activating innate immune cells and inducing inflammation response. What’s more, the combination of D-GalN and LPS was more effective in liver injury.

In view of systemic inflammation, ACLF or ALF shares striking similarities with septic shock, which have been implicated in multi-organ dysfunction. Systemic inflammation results from the secretion of pro-inflammatory cytokines, such as tumor necrosis factor α (TNF-α) interleukin (IL)-1 and IL-6, into peripheral blood. These cytokines are thought to reflect severity of inflammatory response^4^. Human high-mobility group box-1 (HMGB1) is a damage-associated molecular pattern (DAMP) molecule, that can be actively secreted from monocytes/macrophages. Previous studies have shown that extracellular HMGB1 acts to alert host injury thereby resulting in various inflammatory responses, such as pro-inflammatory cytokine production^5^. Particularly, HMGB1 or pro-inflammatory cytokines activate macrophages by activating the NF-κB signaling pathway, which leads to further secretion of pro-inflammatory cytokines^6^. Results from pre-clinical studies show that the secretive inhibition of excessive HMGB1 is an attractive therapeutic strategy for ameliorating liver injury^5^. In addition, recent studies have shown that monocyte dysfunction mediates overexpression of DAMPs from the injured tissue^6^. However, the mechanism through which DAMPs regulate the function of monocyte during LF remains unclear.

LF is a disorder driven by innate immune cells. In fact, monocytes/macrophages reportedly play key roles in innate immune response and liver injury^1,6^. In humans, ~90% of monocytes express high levels of CD14 is termed classical CD14^+^ monocytes, and the numbers of infiltrating monocytes from peripheral blood increase during liver inflammation^7^. In our previous studies, we demonstrated that enhanced cytokine secretion of CD14^+^ monocytes was related to liver inflammation in patients with decompensated liver cirrhosis (DC-LC), whereas deactivation of resident Kupffer cells (KCs) was associated with ALF severity^8,9^. In mice, intrahepatic infiltrated monocyte-derived macrophages were positive for CD11b, and had low levels of the macrophage marker F4/80 (CD11b^hi^F4/80^lo^), which were further divided in Ly-6C^+^ and Ly-6C^−^ subpopulations^10^. Furthermore, CD11b^lo^F4/80^hi^ macrophages represent the KC population in the liver of mice^11^. A study on murine models found accumulation of inflammatory Ly-6C^+^ monocytes in injured liver, exacerbating the damage^12^. On the other hand, blocking of transient recruitment of infiltrated Ly-6C^+^ monocytes resulted in reduced levels of intrahepatic pro-inflammatory cytokines^10^. Overall, these findings indicate that inflammatory response and phenotypic changes in monocyte/macrophages are crucial aspects for understanding liver injury and disease progression. To date, however, no studies have investigated D-GalN/LPS-induced infiltration of monocytes into ALF. Previous studies have shown that autophagy actively participates in and is a regulator or effector of inflammation and innate immunity^13,14^. In fact, a recent study demonstrated that autophagy protected monocytes from inflammation provoked by apoptosis^15^. However, the underlying mechanisms of autophagy and apoptosis in LF in monocytes remain unclear.

T-cell immunoglobulin and mucin domain-containing molecule-3 (Tim-3) is found on the surface of diverse immune cells, including monocytes^16^. Previous studies have shown that a reduction on Tim-3 leads to increased macrophage activation^17^. Results from our previous studies also showed that Tim-3 reduction in monocytes was associated with endotoxemia and inflammatory cytokines in DC-LC^8^. Functionally, Tim-3 is shed from monocytes induced by LPS to produce sTim-3, which plays an inhibitory role in sepsis as well as expression of TNF-α and IL-12^18^. However, no study has reported the role of sTim-3 in regulation of monocyte/macrophage function during LF. The current study, therefore, aimed to investigate whether sTim-3 alters monocyte/macrophage function during LF and elucidate the mechanism of action in vitro and in vivo.

## Results

### Levels of plasma sTim-3 increased in ACLF patients

**Fig. 1:**
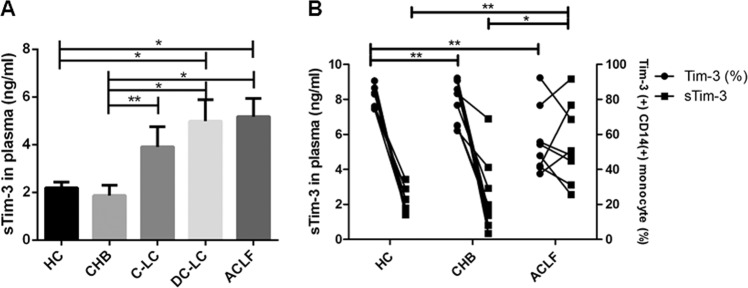
Expression profiles of plasma sTim-3 and membrane Tim-3 in study subjects. **a** Plasma sTim-3 levels in ACLF (*n* = 8), DC-LC (*n* = 8), C-LC (*n* = 8), CHB (*n* = 8), and HC (*n* = 8). Data represent mean ± SEM. **b** The relationship between plasma sTim-3 and membrane Tim-3 in ACLF (*n* = 8), CHB (*n* = 8) and HC (*n* = 8). Data are expressed as plots of individual data. *p < 0.05, ** *p* < 0.01.

To determine the relationship between sTim-3 levels and severity of liver disease, we included and analyzed patients with different stages of liver diseases and HC. Levels of plasma sTim-3 were detected by enzyme-linked immunosorbent assay (ELISA), whereas membrane Tim-3 (mTim-3) in peripheral blood monocytes was analyzed using flow cytometry. The subjects were first divided into ACLF (*n* = 8), DC-LC (*n* = 8), compensated liver cirrhosis (C-LC, *n* = 8), chronic hepatitis B (CHB, *n* = 8) and healthy control (HC, *n* = 8) groups, with analysis revealing an increase in sTim-3 with increase in disease severity (Fig. 1a). Significantly higher sTim-3 levels were recorded in DC-LC and ACLF, than HC and CHB groups. Similarly, sTim-3 levels were also significantly higher in C-LC than CHB group. Conversely, significantly lower mTim-3 levels were recorded in CHB and ACLF than HC group Fig. 1b. Further analysis revealed no correlation between mTim-3 and sTim-3.

### Recombinant sTim-3 affected the various cytokines of monocyte in response to LPS

**Fig. 2:**
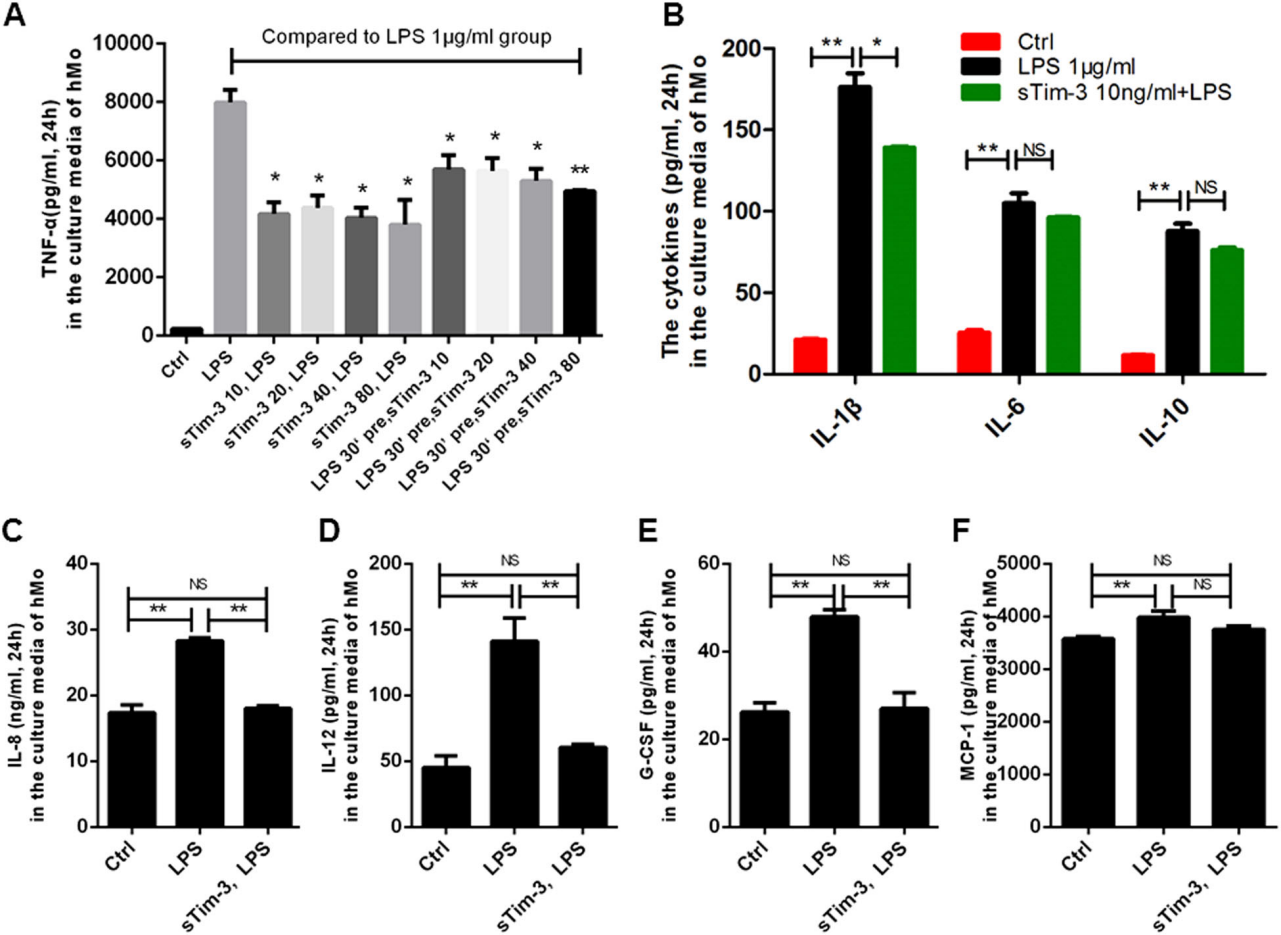
Effect of recombinant sTim-3 on cytokine release in LPS-activated monocytes. Isolated CD14^+^ monocytes from healthy subjects were simultaneously treated with recombinant sTim-3, or 30 min later with 1 μg/ml LPS for 24 h. Cytokine release in monocytes was detected. **a** The levels of TNF-αin the culture media with the sTim-3 concentration of 10, 20, 40, and 80 ng/ml. **b** Levels of IL-1β, 6, and 10 in the culture media supplemented with 10 ng/ml sTim-3. **c**–**f** IL-8, IL-12, G-CSF, and MCP-1 in culture media supplemented with 10 ng/ml sTim-3. Data represent mean ± SEM. *p < 0.05, **p < 0.01; NS, not statistically significant.

Previous studies have shown that plasma sTim-3 plays an inhibitory role in sepsis patients, and is negatively correlated with TNF-α and IL-12^18^. sTim-3 was also reported to inhibit T cell-mediated immune response in tumor rejection model^19^. Based on these studies, we hypothesized that recombinant sTim-3 might inhibit release of pro-inflammatory cytokines from monocytes in response to LPS. Consequently, we measured levels of various cytokines in culture media of monocytes treated with sTim-3 in response to 1 μg/ml LPS. Results showed that sTim-3 significantly inhibited TNF-α expression in a dose-dependent manner, particularly when 10, 20, 40, and 80 ng/ml, were simultaneously added together with LPS or 30 min post LPS stimulation (Fig. 2a). Furthermore, sTim-3 significantly inhibited levels of IL-1β, IL-8, IL-12, and G-CSF, but did not dramatically reduce those of IL-6, IL-10, and chemoattractant protein 1 (MCP-1) (Fig. 2b–f).

### Recombinant sTim-3 infiltrated into monocytes in a dose and time-dependent manner

**Fig. 3:**
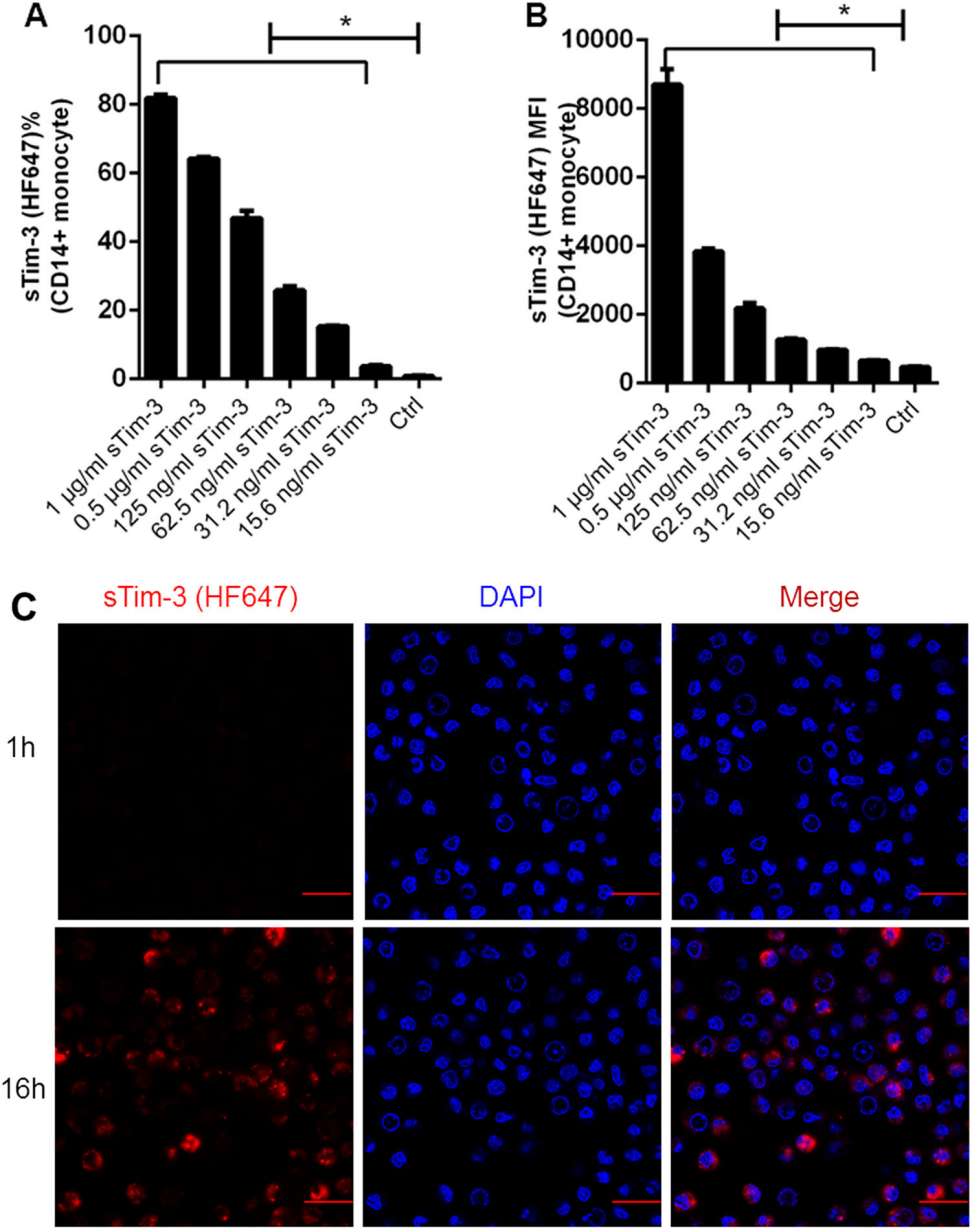
Infiltration of recombinant sTim-3 into monocytes. The CD14^+^ monocytes from healthy subjects were treated with recombinant sTim-3, stained with HiLyte FluorTM 647 (HF647). The infiltrated sTim-3 was then detected. **a** Percentage of infiltrated sTim-3 (HF647) in indicating dose-dependency. **b** Infiltration of sTim-3 (HF647) MFI also showing dose-dependency. **c** Immunofluorescence of the infiltrated sTim-3 (HF647) showing time-dependency (1 and 16 h). Scale bar = 20 μm. Data represent mean ± SEM. *p < 0.05.

Next, we examined how sTim-3 infiltrated into monocytes using flow cytometry and immunofluorescence. Results indicated that recombinant sTim-3 infiltrated into the monocytes, in a dose-dependent manner, by either frequency or MFI at 24-h treatment relative to the control group (Ctrl) (Fig. 3a, b). Representative graphs for frequency and MFI are shown in Figs. S1–1 and S1–2, respectively. Recombinant sTim-3 was labeled with HiLyte Fluor 647, then monocytes treated with the labeled sTim-3 for 1 and 16 h. Immunofluorescence result showed that the labeled sTim-3 infiltrated into the cytoplasm of the monocytes at 16 h, but not at 1 h (Fig. 3c).

### Recombinant sTim-3 inhibited HMGB1 translocation and release of monocytes in response to LPS

**Fig. 4:**
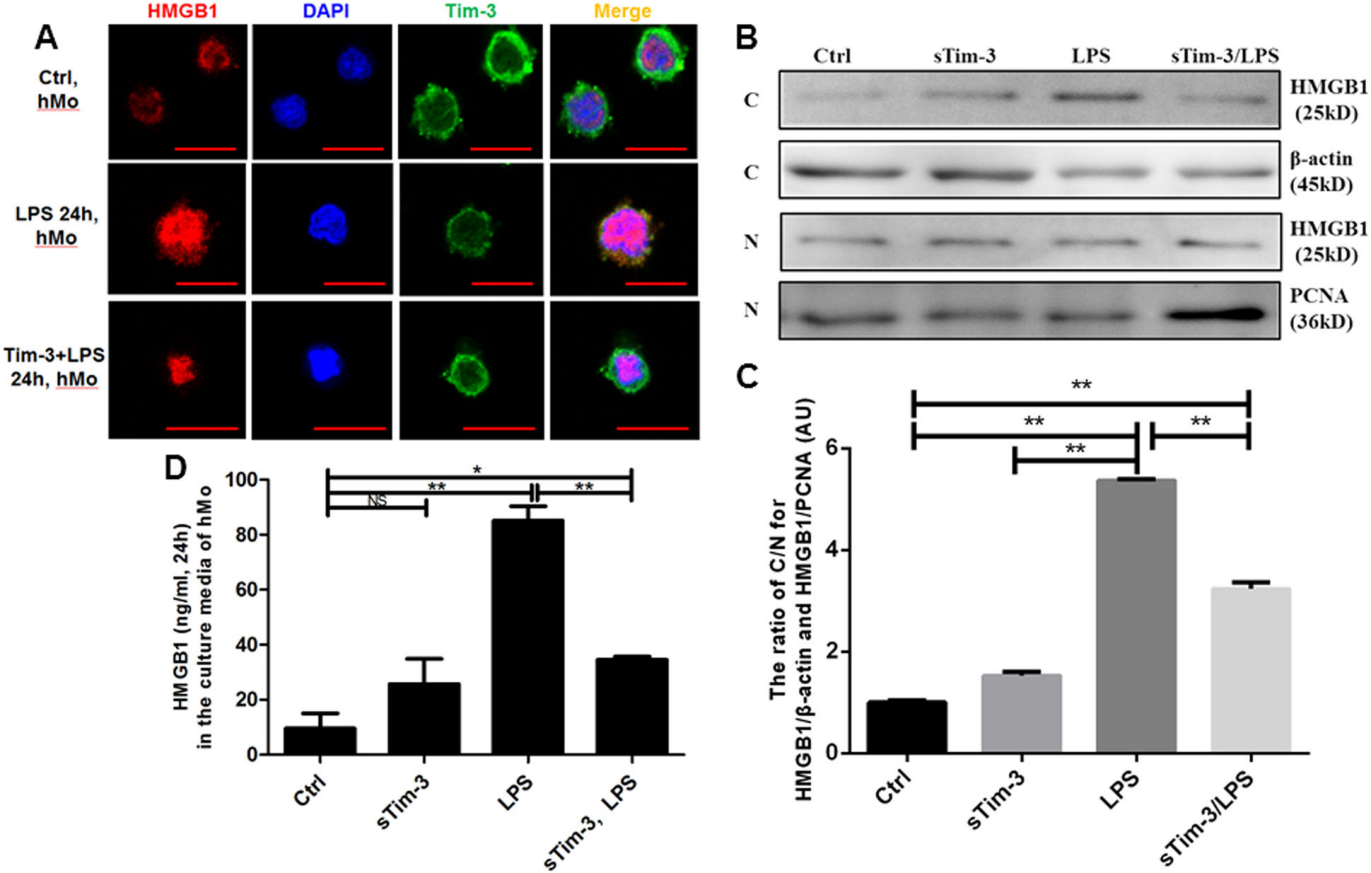
Inhibition of HMGB1 translocation and release of activated monocytes by recombinant sTim-3. CD14^+^ monocytes from healthy subjects were simultaneously treated with 10 ng/ml recombinant sTim-3 and 1 μg/ml LPS for 24 h. Inhibition of HMGB1 translocation was detected by immunofluorescence and western blot assay, whereas HMGB1 release was detected by ELISA. **a** Representative immunofluorescence for HMGB1 and sTim-3 in monocytes. Scale bar = 10 μm. **b** Representative protein blots for HMGB1 in monocytes. PCNA: proliferating cell nuclear antigen; C: cytoplasm; N: nucleus. **c** The ratio of C/N for HMGB1 protein levels in monocytes. **d** Levels of HMGB1 in culture media. Data are expressed as mean ± SEM. **p < 0.01; NS, not statistically significant.

Immunofluorescence results revealed significantly higher cytoplasmic HMGB1 fluorescence in the LPS group, evidenced by brighter signals, than in Ctrl and sTim-3+ LPS groups (Fig. 4a). In addition, Western blots confirmed significantly lower expression ratio of cytoplasm/nuclear HMGB1 in sTim-3+ LPS-treated cells than those in the LPS group (Fig. 4b, c). Furthermore, we found significantly lower levels of HMGB1 expression in supernatants of cells exposed to LPS and sTim-3 for 24 h compared to those in the LPS group (Fig. 4d). Based on previous research that has shown that LPS induces monocyte activation through multiple signaling pathways, we determined whether sTim-3 affected the NF-κB pathway related to HMGB1 in response to LPS in vitro. Results showed that LPS stimulation, at 1 h, promoted NF-κB phosphorylation, although no differences were observed relative to sTim-3 group (Fig. S2).

### Recombinant sTim-3 promoted autophagy in monocytes

**Fig. 5:**
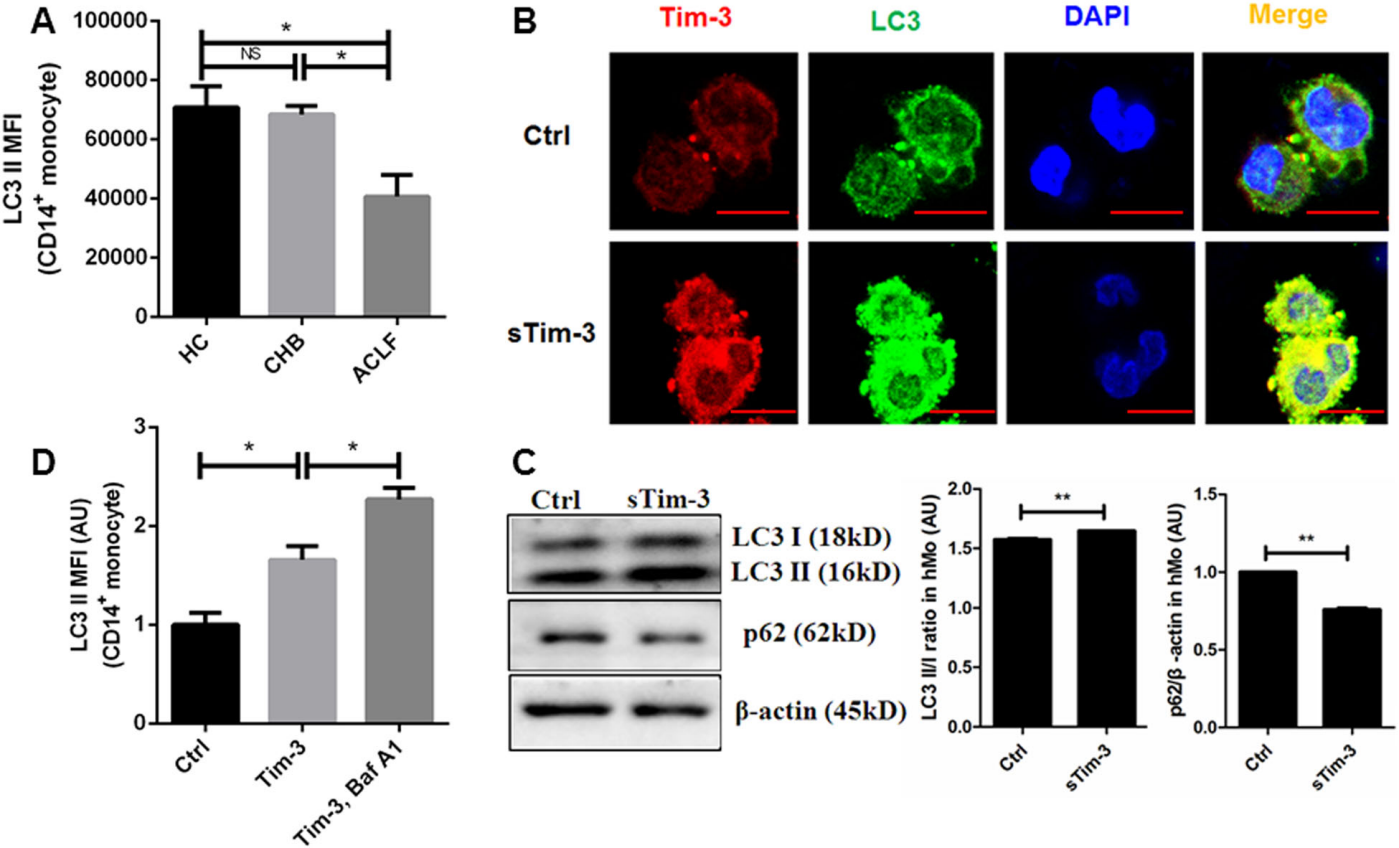
Inhibition of monocyte activation by recombinant sTim-3 via autophagy induction. Autophagy levels in CD14^+^ monocytes from ACLF (*n* = 5), CHB (*n* = 4), and HC (*n* = 5) were detected by flow cytometry for LC3 II. The isolated healthy CD14^+^ monocytes were treated with 10 ng/ml recombinant sTim-3 for 6 h. The autophagy levels were detected by immunofluorescence or western blots for LC3. For the autophagy flux detection, the monocytes were co-treated with 10 ng/ml recombinant sTim-3 and 100 nM Baf A1 and were detected by flow cytometry for LC3 II. **a** Autophagy levels of CD14^+^ monocytes from three groups of study subjects. **b** Representative immunofluorescence of Tim-3 and LC3 for autophagy induction of healthy CD14^+^ monocytes. Scale bar = 10 μm. **c** Western blots indicating LC3 II/I ratio in healthy CD14^+^ monocytes. **d** Detection of autophagy flux in healthy CD14^+^ monocytes. Data are expressed as mean ± SEM. *p < 0.05, **p < 0.01; NS, not statistically significant.

Autophagy in the monocytes was lower in ACLF than CHB patients and the HC groups (Fig. 5a). Based on these findings, we investigated whether sTim-3 affected autophagy in the monocytes, as a possible mechanism of LPS-mediated inhibition of monocyte activation. In fact, previous studies have reported that intracellular HMGB1 is co-localized with LC3 and mediated by autophagy^20^. Therefore, we first observed LC3 puncta of monocytes to evaluate the autophagic activity affected by recombinant sTim-3 through immunofluorescence, and found that brighter LC3 puncta signals in the sTim-3 than the ctrl groups. Moreover, LC3 was colocalized with Tim-3 (Fig. 5b). To further confirm that sTim-3 promotes autophagy, we performed a Western blot analysis using LC3 and p62 antibodies. Results indicated that sTim-3 increased the amount of LC3-II/I ratio and decreased that of p62 (Fig. 5c). Analysis of autophagic flux confirmed autophagy promotion of sTim-3 by LC3 II (Fig. 5d) and Cyto-ID flow cytometry in monocytes using *Baf A1* (Fig. S3). Overall, these results supported the notion that recombinant sTim-3 promoted autophagosome formation but did not block autophagic flux.

### Recombinant sTim-3 alleviated D-GalN/LPS-induced hepatic damage

**Fig. 6:**
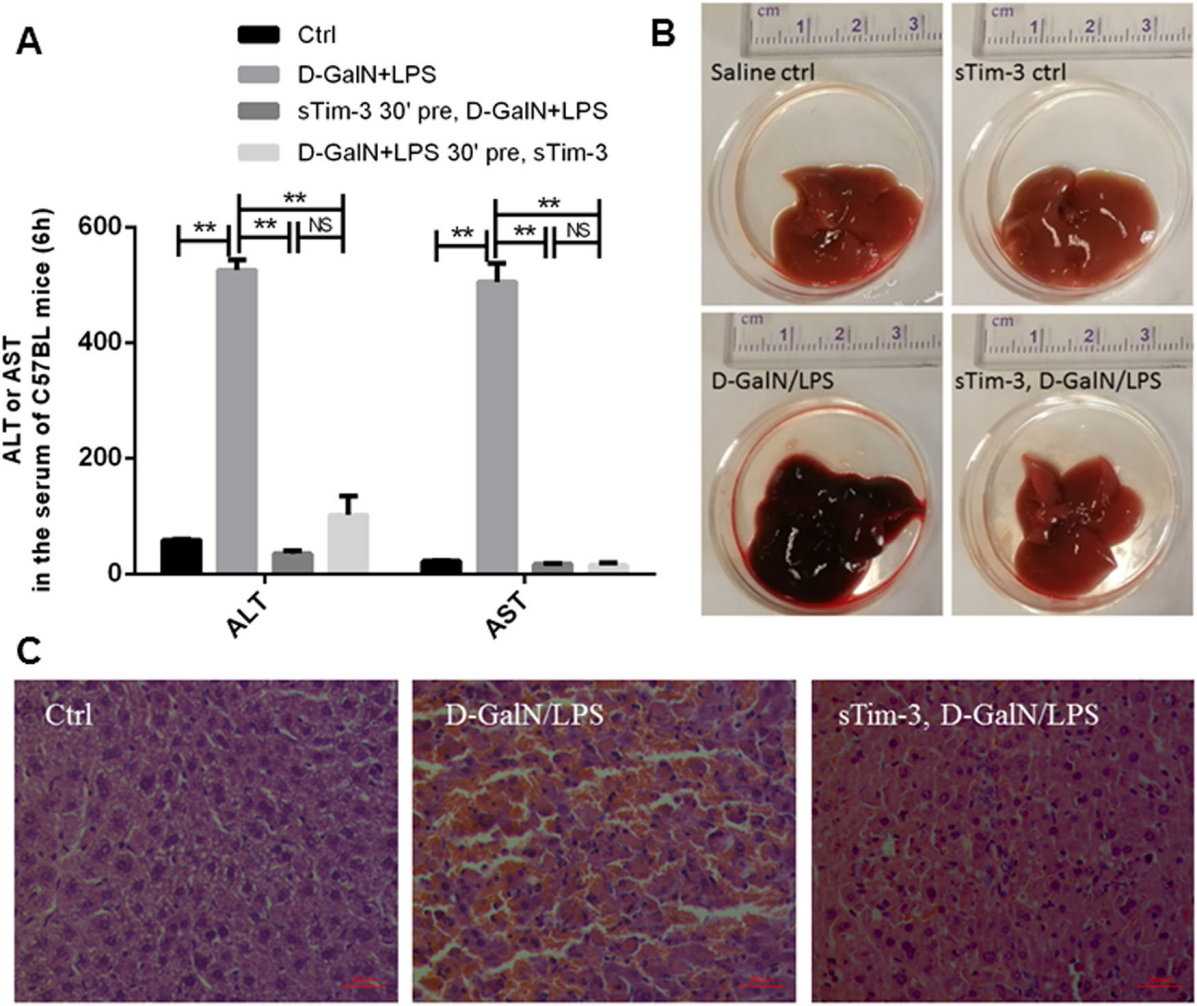
Recombinant sTim-3 alleviated hepatic damage induced by D-GalN/LPS. Male WT C57BL/6 J mice were intraperitoneally administered with 500 mg/kg body weight D-GalN in combination with 5 μg/kg body weight LPS or saline as the control. The treatment group was given in the tail vein 10 μg/kg body weight recombinant sTim-3 30 min after or before induction of liver damage. **a** Serum levels of ALT and AST measured 6 h after D-GalN/LPS challenge. **b** Macro appearance of representative liver samples in all groups 6 h after D-GalN/LPS challenge. Treated mice were administered with recombinant sTim-3 30 min before induction of liver damage. **c** Representative H&E staining of liver samples. Scale bar = 100 μm. Data are expressed as mean ± SEM. **p < 0.01; NS, not statistically significant.

Previous studies have reported that ALF is characterized by profound activation of monocytes^6^, whereas in the current study, we found that sTim-3 inhibited monocyte activation. Based on these findings, we determined whether sTim-3 could improve hepatic damage in D-GalN/LPS mice. Our results showed increased levels of alanine aminotransferase (ALT) and aspartate transaminase (AST), widely accepted serum markers, increased at 6 h after D-GalN/LPS administration (Fig. 6a). Conversely, 30 min pre- or post-treatment with sTim-3 (10 μg/kg) resulted in low ALT and AST levels (Fig. 6a). Furthermore, H&E staining results further confirmed that sTim-3 alleviated liver injury in D-GalN/LPS mice (Fig. 6b, c).

### Recombinant sTim-3 regulated monocyte/macrophage populations

**Fig. 7:**
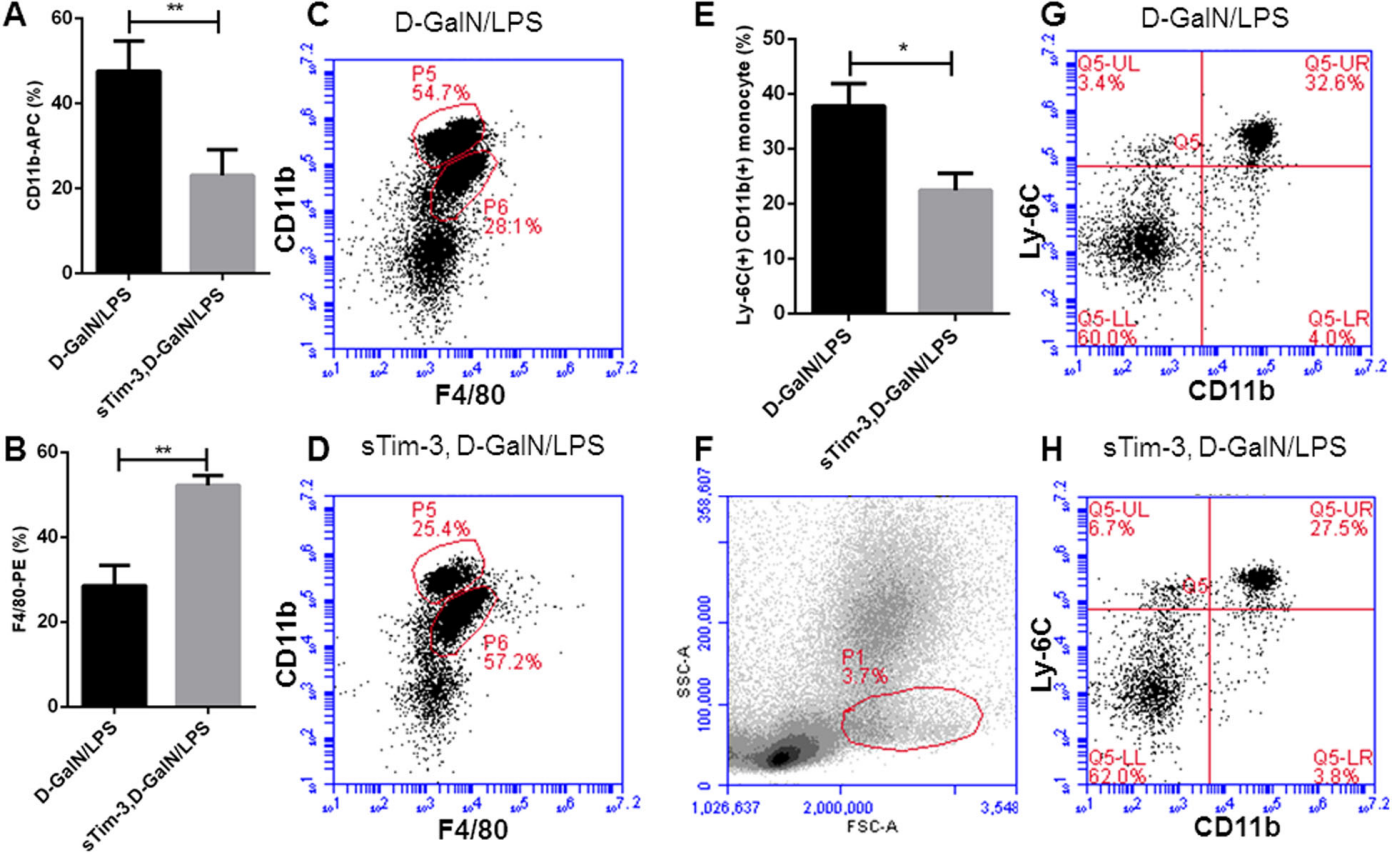
Effect of recombinant sTim-3 on monocyte/macrophage populations in the liver and blood. There were two groups: 1) D-GalN/LPS group; 2) recombinant sTim-3-treated D-GalN/LPS group. The liver injury of mice was induced by D-GalN/LPS as Fig. 6. 30 min after the liver damage was induced, the treatment group was given in the tail vein 10 μg/kg body weight recombinant sTim-3. Six hours after the liver damage was induced, liver samples and blood in all groups were collected, and surface F4/80 and CD11b of monocyte/macrophages in the liver were analyzed by flow cytometry. **a**–**d** Percentage of CD11b^hi^F4/80^lo^ monocyte-derived and CD11b^lo^F4/80^hi^ tissue-resident macrophages in the liver. **e**–**h** The percentage of inflammatory Ly-6C(+)CD11b(+) monocytes in blood. *p < 0.05; **p < 0.01.

Relative surface expression of CD11b and F4/80 can be used to distinguish infiltrating monocytes and tissue-resident macrophages. Previous studies have shown that an increase in the number of monocytes may contribute to inflammatory damage in D-GalN/LPS mice^21^. In this study, we analyzed populations of CD11b and F4/80 in mice livers using flow cytometry, and found that recombinant sTim-3 reduced the percentage of CD11b^hi^F4/80^lo^ monocyte-derived, but increased CD11b^lo^F4/80^hi^ tissue-resident macrophages in the D-GalN/LPS-induced liver (Fig. 7a–d). Since inflammatory Ly-6C^+^ monocytes are the primary source of TNF-α and critical mediators in liver injury^10,22^, we next detected the percentage of inflammatory Ly-6C(+)CD11b(+) monocytes in the blood and found that sTim-3 significantly lowered the percentage of these inflammatory monocytes (Fig. 7e–h).

### Recombinant sTim-3 influenced autophagy and apoptosis of monocytes/macrophages in the liver

**Fig. 8:**
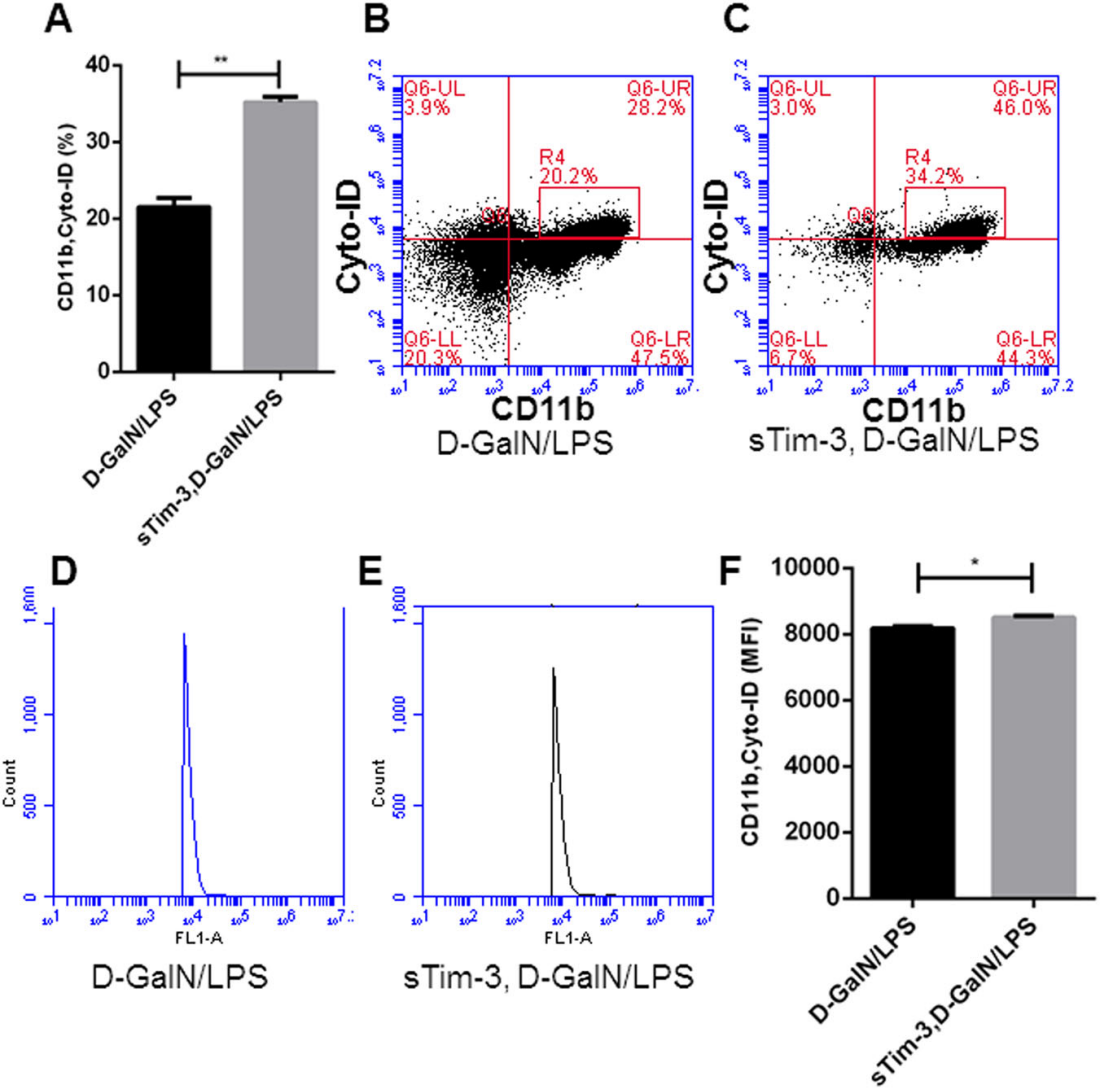
Effect of sTim3 on autophagy in hepatic monocytes. This was performed on the liver injury and the treatment groups as shown in Fig. 8. 6 h after D-GalN/LPS challenge, liver samples in two kinds of groups were collected. The autophagy levels of hepatic CD11b monocytes with Cyto-ID dye were analyzed by flow cytometry. **a**–**c** Rate of autophagy in CD11b monocytes. **d**–**f** Mean fluorescence intensity (MFI) of autophagy in CD11b monocytes. *p < 0.05; **p < 0.01.

Previous studies have shown that increased autophagy prevents apoptosis in primary hepatocytes, thus protecting them from liver inflammation injury of ALF mice induced by D-GalN/LPS^2,23^. In addition, other studies have reported autophagy-induced inhibition of inflammation and apoptosis in monocytes^15,24^. In the current study, we analyzed autophagy levels of CD11b(+) monocyte-derived macrophages in the liver and found higher levels in the sTim-3 compared to the D-GalN/LPS group, as evidenced by percentage and mean fluorescence intensities (Fig. 8a–f). Moreover, we found a lower apoptosis rate of CD11b(+) monocytes in the blood of sTim-3 relative to the D-GalN/LPS group (Fig. S4A–E).

## Discussion

In this study, we demonstrated that sTim-3 inhibited production of inflammatory mediators and regulated the functional status as well as populations of monocytes/macrophages, possibly through autophagy. Taken together, these findings confirmed that monocytes/macrophages play an important role in LF. Previous studies have shown that reduction of Tim-3 in monocytes is accompanied by sepsis severity, and sTim-3 levels are elevated in septic shock, which generates an inhibitory role during sepsis-induced immunosuppression^18^. Other studies have demonstrated that sTim-3 shedding from monocytes is a biomarker for immune exhaustion^25^, and is subsequently involved in progression of HBV-related liver disease^26^. Results of the current study showed significantly lower mTim-3 of monocytes in ACLF patients, whereas plasma sTim-3 increased during disease progression, which is consistent with the previous study^26^. LF is characterized by profound activation of monocytes, although only a handful of studies have investigated relationships between the function of circulating monocytes and inflammatory mediator production in LF^3^. When compared to healthy controls, monocytes in ALF were found to produce high levels of TNF-α, suggesting that they are an important source of this cytokine^3^. In the current study, we found that sTim-3 inhibited secretion of TNF-α, in monocytes induced by LPS, in a dose-dependent manner. Furthermore, previous studies have shown that stimulation of many pro-inflammatory cytokines, including IL-1, IL-6, and MCP-1 in KCs, by LPS could trigger hepatocyte cell death and cause ALF^27^. In the present study, sTim-3 significantly inhibited IL-1β, IL-8, IL-12, and G-CSF, but not IL-6, IL-10, and MCP-1. We also confirmed that sTim-3 infiltrated into the cells, in a dose-dependent manner and at later times. These findings conclude that sTim-3 and mTim-3 are associated with disease progression, and may be related to inhibition of various cytokines in activated monocytes.

Previous studies have reported that LPS and HMGB1 can induce systemic inflammatory response syndrome (SIRS). Particularly, HMGB1 has been implicated in cytokine production^28^. In our previous research, we demonstrated that the anti-inflammatory activity of shikonin in macrophages is through inhibition of HMGB1 translocation, and this is associated with the NF-κB signaling pathway^28^. Furthermore, Tim-3 signaling inhibited LPS-TLR4-mediated NF-kB activation^17^. Based on these findings, we investigated the mechanism of cytokine secretion from HMGB1 and NF-κB, and found that sTim-3 inhibited translocation and release of HMGB1 in LPS-stimulated monocytes, but did not affected phosphorylation of the NF-κB signaling pathway. Therefore, further investigation is needed to fully understand the mechanism of HMGB1 modulation^5^. Previous studies have demonstrated that HMGB1 release is mediated by autophagy, a protective process for cellular response to injury and inflammation. In addition, Tanshinone iia was shown to stimulate cytoplasmic HMGB1 protein co-localized with LC3 punctate^20^. In the current study, our results showed lower rates of autophagy in monocytes of ACLF patients, but this was promoted by sTim-3. These results indicated that inhibition of monocyte activation may be through autophagy promotion. In addition, compared to the control group, we found brighter LC3 puncta signals and co-location with Tim-3 in sTim-3-treated monocytes. Furthermore, our results confirmed increased expression of LC3-II/I, and decreased p62. Our result also showed that autophagic flux was not blocked. Collectively, these findings support the notion that sTim-3 mediated an increase in autophagy levels in monocytes, which inhibits production of inflammatory mediators.

Next, we investigated whether sTim-3 alleviated liver damage in a murine D-GalN/LPS model. Results from organ injury markers and H&E staining confirmed liver protection by sTim-3 in vivo. Previous studies have suggested that a correlation between the number of monocytes/macrophages with inflammatory response and disease severity^29^. Particularly, KCs were found to produce various modulatory factors with anti-inflammatory function^30,31^. On the other hand, significantly low percentages of KCs were recorded in an ALF murine model^9^, with depletion of KCs found to lead to an increase in susceptibility to acetaminophen-induced liver injury^31^. In the current study, our results showed high percentages of CD11b^lo^F4/80^hi^ macrophages (KCs) in ALF mice, which may indicate a potential anti-inflammatory mechanism for sTim-3. However, there are also contrast studies. The number of hepatic KCs in Per1 deletion of D-GalN/LPS mice significantly increased, thereby inducing elevated levels of pro-inflammatory cytokines^21^. Evidence from acetaminophen toxicity, in human and animal models, suggests that influx of monocytes from circulation contributes to increase in hepatic macrophages^11,32^. In mice, Ly-6C^hi^ monocytes are infiltrated during early stages, to the inflammatory area, and exhibit pro-inflammatory profiles^33,34^. Furthermore, results of the present study showed that sTim-3 reduced percentages of hepatic CD11b^hi^F4/80^lo^ population as well as pro-inflammatory Ly-6C(+)CD11b(+) monocytes in the blood in D-GalN/LPS mice^35^. In addition, some ALF studies have reported that depletion of recruited monocyte-derived macrophages has a protective effect on liver injury^36^. However, the actual function of the recruited macrophages, particularly macrophage depletion impaired resolution responses in ALF, remain unclear owing to contradictory evidence^37^.

Previous studies have reported repression of autophagy in D-GalN/LPS mice, with its activation found to confer protection against liver injury in ALF mice models^38^. Furthermore, autophagy activation was found to mediate reduction of inflammatory response in macrophages or healthy monocytes by inhibiting activation of TNF-α secretion or inflammasome^24,39^. In the current study, we investigated whether sTim-3 caused induction of autophagy in monocytes/macrophages by analyzing hepatic CD11b (+) cell population using flow cytometry. Our results confirmed that sTim-3 increased autophagy levels of CD11b^+^ cells in D-GalN/LPS mice, but reduced the rate of apoptosis in CD11b(+) monocytes. These results were consistent with previous evidence which demonstrated that induction of autophagy inhibited apoptosis in monocytes^24^.

The current study was limited by a lack of autophagy inducers, such as rapamycin, as a positive control for in vivo and in vitro experiments. Nevertheless, our results confirmed that induction of autophagy mediated a reduction of inflammatory response in monocytes and alleviated liver injury in ALF. In addition, results from several techniques demonstrate that sTim-3 promotes autophagy in monocytes/macrophages.

Collectively, results of the present study confirmed a change in levels of sTim-3 in patients and demonstrated that recombinant sTim-3 inhibited LPS-induced inflammatory response in monocytes. In addition, HMGB1 release and autophagy induction may be the potential mechanism in vitro study. In vivo experiments have showed that sTim-3 affects autophagy and apoptosis of monocytes/macrophages, resulting in an effect in percentages of cell phenotypes. Taken together, these results provide new insights into regulation of the function of monocytes/macrophages in LF, which could be important for future development of treatment strategies against inflammatory response. However, better understanding of the potential mechanisms of liver damage is needed to guide development of immunomodulatory strategies for clinical management of LF.

## Materials and methods

### Study subjects

We retrospectively recruited a total of 32 patients at the outpatient or inpatient service of the Department of Infectious Diseases in the First Affiliated Hospital of Zhejiang University. Eight (8) patients had ACLF, 8 exhibited DC-LC, 8 had C-LC, whereas 8 others had CHB. Individual patients were diagnosed according to the guidelines of the Chinese Group on the Study of Severe Hepatitis B^40^. The patients with ascites, upper gastrointestinal hemorrhage, and hepatic encephalopathy were diagnosed as DC-LC, whereas those with cirrhosis were diagnosed as C-LC. A total of 44 HC were also simultaneously recruited from the physical examination center of our hospital. Individuals were excluded if: (1) they were younger than 18 and more than 80-years old; (2) were co-infected with other viruses such as human immunodeficiency virus or hepatitis C virus (3) had autoimmune diseases; and (4) had a malignancy. Demographic and clinical characteristics of all subjects included in the study are listed in Supplementary Tables 1 and 2. The experimental protocol used herein and in the following animal studies were approved by the Ethics Committee of Zhejiang University.

### CD14^+^ monocyte isolation and treatment

A combination of density centrifugation and CD14-microbeads was used to isolate human CD14^+^ monocytes as previously described^8^. In brief, peripheral blood mononuclear cells were first separated from fresh anti-coagulation blood by Ficoll-Hypaque density centrifugation, then CD14^+^ monocytes isolated using a kit containing CD14-microbeads (Cat. No. 130-117-337. Miltenyi, Bergisch Gladbach, Germany). The prepared monocytes, with a purity of 90%, were used in subsequent experiments. Recombinant human sTim-3 (Cat.No.10390-H08H. Sino Biological Inc., Beijing, China) was used to treat human CD14^+^ monocytes.

### Measurement of sTim-3 and cytokines

Expression levels of sTim-3 in plasma of enrolled subjects were determined using the Human TIM-3 Quantikine ELISA kit (Cat. No. DTIM30. RD Biosciences, Minnesota, USA) according to the manufacturer’s instructions. Concentrations of TNF-α, IL-1β, IL-6, and IL-10 in the culture media were measured using ELISA kits (Cat. No. DTA00D, DLB50, D6050, D1000B, RD Biosciences, Minnesota, USA), whereas levels of IL-8, IL-12, G-CSF, and MCP-1 in the culture media were detected using the Bio-Plex Pro^TM^ Human Cytokine Standard 27-Plex, Group I kit (Cat. No. 171-D50001. Bio-Rad, California, USA)^41^.

### Determination of cell surface Tim-3 and infiltration of sTim-3 into monocytes

Surface Tim-3 of CD14^+^ monocytes from ACLF, CHB, and HC individuals were detected by flow cytometry. Summarily, recombinant human sTim-3 (Cat.No.10390-H08H-50. Sino Biological Inc., Beijing, China) was first stained with HF647, using the Ab-10 Rapid HiLyte FluorTM 647 Labeling Kit (Cat. No. LK36. Dojindo, Shanghai, China) according to the manufacturer’s instructions. Percentages and mean fluorescence intensities (MFI) of infiltrated sTim-3 (HF647) into the monocytes were then analyzed by flow cytometry. Imaging of infiltrated sTim-3 (HF647) was done by laser scanning confocal microscopy (Carl Zeiss, Oberkochen, German).

### Detection of HMGB1 translocation and release in monocytes

Fluorescence of HMGB1 in CD14^+^ monocytes was detected by confocal microscopy (Carl Zeiss, Oberkochen, German), whereas the ratio of cytoplasm/nuclear HMGB1 in CD14^+^ monocytes was measured by Western blot analysis as previously described^28^, using a HMGB1 antibody from Abcam (Cat. No. ab18256, Massachusetts, USA). HMGB1 levels in the culture media of CD14^+^ monocytes were measured by an ELISA kit (Cat. No. SEA399Hu. USCN Business Co., Ltd. Wuhan, China) according to the manufacturer’s instructions.

### In vitro analysis of autophagy levels in recombinant sTim-3-influenced monocytes

Autophagy was detected by flow cytometry, fluorescent microscopy and Western blot analysis^42–45^. In brief, autophagy levels in monocytes from ACLF, CHB, and HC individuals were measured by flow cytometry for LC3 II MFI. This method was further used to detect autophagy flux using Cyto-ID (Cat. No. ENZ-51031-K200. Enzo, Farmingdale, New York, USA) in monocytes treated with recombinant sTim-3 and bafilomycin A1 (Cat. No. 1334. Baf A1, Tocris, Missouri, USA). Colocalization of LC3 and Tim-3 was analyzed by fluorescent microscopy. The used antibodies contained primary LC3 antibody (Cat. No. L7543, Sigma, St. Louis, USA) and the corresponding Alexa Fluor 488-conjugated anti- rabbit secondary Antibody (Cat. No. A31620. Molecular probes, Massachusetts, USA), primary anti-Tim-3 antibody (Cat. No. ab268138, Abcam, Massachusetts, USA) and the corresponding Alexa Fluor 568-conjugated anti-mouse secondary Antibody (Cat. No. A-11031. Molecular probes, Massachusetts, USA). Autophagy inducer of sTim-3 was detected by Western blot analysis, using primary antibodies p62 (Cat. No. PM045. MBL International, Nagoya, Japan) and LC3.

### Detection of NF-κB phosphorylation in cultured cells

The impact of recombinant sTim-3 on levels of NF-κB phosphorylation in LPS-activated (1 μg/ml) CD14+ monocytes was measured using the protocol described in the InstantOne ELISA assay Kit (Cat. No. 85–86083–11. eBioscience, California, USA). Measurements were performed at 0.5, 1.0 and 1.5 h.

### Evaluation of acute LF induced by D-GalN/LPS and liver injury

Male C57BL/6 mice, aged between 6–8-week old, were intraperitoneally injected with 500 mg/kg of D-GalN (Cat. No. G0500. Sigma, California, USA) and LPS (5 μg/kg; Cat. No. L4391. *Escherichia coli*, O111: B4; Sigma, California, USA), to induce ALF. Recombinant mouse sTim-3 (10 μg/kg) (Cat.No.51152-M08H. Sino Biological Inc., Beijing, China) was intravenously administrated to the mice, 30 min before or after the injection of D-GalN/LPS, with the control group administrated with the same volume of normal saline. The mice were randomly divided using random numbers table into three groups (*n* = 5) comprising: (a) saline control; (b) D-GalN/LPS; and (c) recombinant sTim-3-treated D-GalN/LPS. The mice were sacrificed, 6 h following D-GalN/LPS injection, then their livers and blood samples were collected for further analysis. No blinding was done.

Liver injury was assessed using ALT or AST, as injury markers (No.20162404023, No.20162404022. DRI-CHEM 4000ie, Fujifilm, Japan). Histological analysis of stained tissues was also performed according to previously described methods^23^.

### Analysis of monocytes/macrophages in the liver

We divided the mice into two groups (*n* = 5) using random numbers table, D-GalN/LPS and recombinant sTim-3-treated D-GalN/LPS, then isolated murine hepatic mononuclear cells containing monocytes/macrophages according to a previously reported protocol^8^. No blinding was done. To analyze the cells, we first stained them with APC-labeled anti-CD11b (Cat. No. 101211. Biolegend, California, USA), PE-labeled anti-F4/80 (Cat. No. 123109. Biolegend, California, USA) and PerCP-Cy5.5-labeled anti- Ly-6C (Cat. No. 128011. Biolegend, California, USA) antibodies, then washed them with PBS. Levels of the above cell surface markers were then detected with the Accuri C6 cytometer (Accuri, BD, USA), whereas percentages of apoptotic cells were determined using Annexin V-FITC Fluorescence Microscopy Kit (Cat. No. 550911. BD, New Jersey, USA), followed by CFlow software analysis (Accuri C6 cytometer, New Jersey, BD, USA).

### Statistical analysis

Data were presented as means ± standard errors of the means (SEM). The variance between the groups is similar. Differences between groups were determined using a two-tailed Student’s *t*-test by SPSS 17.0 for Windows (SPSS Inc, Chicago, IL, USA). **p < 0.01 or *p < 0.05 (two-tailed) was considered statistically significant. The experiments were repeated three times at least.

## Supplementary information

Supplementary Figure 1

Supplementary Figure 2

Supplementary Figure 3

Supplementary Figure 4

Supplementary figure legends

Supplementary Tables 1 and 2

## References

[1] Murray KF, Hadzic N, Wirth S, Bassett M, Kelly D (2008). Drug-related hepatotoxicity and acute liver failure. J. Pediatr. Gastroenterol. Nutr..

[2] Shi Y (2015). Acute-on-chronic liver failure precipitated by hepatic injury is distinct from that precipitated by extrahepatic insults. Hepatology.

[3] Li J (2018). AMP-activated protein kinase agonist N(6)-(3-hydroxyphenyl)adenosine protects against fulminant hepatitis by suppressing inflammation and apoptosis. Cell Death Dis..

[4] Antoniades CG (2006). Reduced monocyte HLA-DR expression: a novel biomarker of disease severity and outcome in acetaminophen-induced acute liver failure. Hepatology.

[5] Khambu B, Yan S, Huda N, Yin XM (2019). Role of high-mobility group box-1 in liver pathogenesis. Int. J. Mol. Sci..

[6] Possamai LA, Thursz MR, Wendon JA, Antoniades CG (2014). Modulation of monocyte/macrophage function: a therapeutic strategy in the treatment of acute liver failure. J. Hepatol..

[7] Liaskou E (2013). Monocyte subsets in human liver disease show distinct phenotypic and functional characteristics. Hepatology.

[8] Shi Y (2015). Decreased Tim-3 expression is associated with functional abnormalities of monocytes in decompensated cirrhosis without overt bacterial infection. J. Hepatol..

[9] Yang Q (2013). The role of intracellular high-mobility group box 1 in the early activation of Kupffer cells and the development of Con A-induced acute liver failure. Immunobiology.

[10] Baeck C (2014). Pharmacological inhibition of the chemokine C-C motif chemokine ligand 2 (monocyte chemoattractant protein 1) accelerates liver fibrosis regression by suppressing Ly-6C(+) macrophage infiltration in mice. Hepatology.

[11] Holt MP, Cheng L, Ju C (2008). Identification and characterization of infiltrating macrophages in acetaminophen-induced liver injury. J. Leukoc. Biol..

[12] Tacke F, Zimmermann HW (2014). Macrophage heterogeneity in liver injury and fibrosis. J. Hepatol..

[13] Deretic V (2012). Autophagy as an innate immunity paradigm: expanding the scope and repertoire of pattern recognition receptors. Curr. Opin. Immunol..

[14] Into T, Inomata M, Takayama E, Takigawa T (2012). Autophagy in regulation of Toll-like receptor signaling. Cell Signal.

[15] Kamalakannan V, Shiny A, Babu S, Narayanan RB (2015). Autophagy protects monocytes from Wolbachia heat shock protein 60-induced apoptosis and senescence. PLoS Negl. Trop. Dis..

[16] Anderson AC (2007). Promotion of tissue inflammation by the immune receptor Tim-3 expressed on innate immune cells. Science.

[17] Yang X (2013). T cell Ig mucin-3 promotes homeostasis of sepsis by negatively regulating the TLR response. J. Immunol..

[18] Ren F (2015). Plasma soluble Tim-3 emerges as an inhibitor in sepsis: sepsis contrary to membrane Tim-3 on monocytes. Tissue Antigens.

[19] Geng H (2006). Soluble form of T cell Ig mucin 3 is an inhibitory molecule in T cell-mediated immune response. J. Immunol..

[20] Zhang Y (2012). Tanshinone IIA sodium sulfonate facilitates endocytic HMGB1 uptake. Biochem Pharm..

[21] Wang T (2016). PER1 prevents excessive innate immune response during endotoxin-induced liver injury through regulation of macrophage recruitment in mice. Cell Death Dis..

[22] Ocuin LM (2012). Nilotinib protects the murine liver from ischemia/reperfusion injury. J. Hepatol..

[23] Lin X (2018). Liver-specific deletion of Eva1a/Tmem166 aggravates acute liver injury by impairing autophagy. Cell Death Dis..

[24] Sanjurjo L (2015). The human CD5L/AIM-CD36 axis: a novel autophagy inducer in macrophages that modulates inflammatory responses. Autophagy.

[25] Zilber E (2019). Soluble plasma programmed death 1 (PD-1) and Tim-3 in primary HIV infection. AIDS.

[26]  Li F (2018). Highly elevated soluble Tim-3 levels correlate with increased hepatocellular carcinoma risk and poor survival of hepatocellular carcinoma patients in chronic hepatitis B virus infection. Cancer Manag. Res..

[27] Schmöcker C (2007). Omega-3 fatty acids alleviate chemically induced acute hepatitis by suppression of cytokines. Hepatology.

[28] Yang Y (2014). Shikonin inhibits the lipopolysaccharide-induced release of HMGB1 in RAW264.7 cells via IFN and NF-κB signaling pathways. Int. Immunopharmacol..

[29] Bernsmeier C (2015). Patients with acute-on-chronic liver failure have increased numbers of regulatory immune cells expressing the receptor tyrosine kinase MERTK. Gastroenterology.

[30] Borst K (2018). Type I interferon receptor signaling delays Kupffer cell replenishment during acute fulminant viral hepatitis. J. Hepatol..

[31] Ju C (2002). Protective role of Kupffer cells in acetaminophen-induced hepatic injury in mice. Chem. Res. Toxicol..

[32] Antoniades CG (2012). Source and characterization of hepatic macrophages in acetaminophen-induced acute liver failure in humans. Hepatology.

[33] Gordon S, Taylor PR (2005). Monocyte and macrophage heterogeneity. Nat. Rev. Immunol..

[34] Ingersoll MA (2010). Comparison of gene expression profiles between human and mouse monocyte subsets. Blood.

[35] Ramachandran P (2012). Differential Ly-6C expression identifies the recruited macrophage phenotype, which orchestrates the regression of murine liver fibrosis. Proc. Natl Acad. Sci. USA.

[36] Dambach DM, Watson LM, Gray KR, Durham SK, Laskin DL (2002). Role of CCR2 in macrophage migration into the liver during acetaminophen-induced hepatotoxicity in the mouse. Hepatology.

[37] You Q (2013). Role of hepatic resident and infiltrating macrophages in liver repair after acute injury. Biochem. Pharm..

[38] Ren F (2016). Inhibition of glycogen synthase kinase 3β promotes autophagy to protect mice from acute liver failure mediated by peroxisome proliferator-activated receptor α. Cell Death Dis..

[39] Zhai Y (2018). TNFAIP3-DEPTOR complex regulates inflammasome secretion through autophagy in ankylosing spondylitis monocytes. Autophagy.

[40] Wu T (2018). Development of diagnostic criteria and a prognostic score for hepatitis B virus-related acute-on-chronic liver failure. Gut.

[41] Bonacini M (2020). Cytokine profiling in aqueous humor samples from patients with non-infectious uveitis associated with systemic inflammatory diseases. Front. Immunol..

[42] Demishtein A, Porat Z, Elazar Z, Shvets E (2015). Applications of flow cytometry for measurement of autophagy. Methods.

[43] van Loosdregt J (2016). Increased autophagy in CD4(+) T cells of rheumatoid arthritis patients results in T-cell hyperactivation and apoptosis resistance. Eur. J. Immunol..

[44] Kozako T (2015). Novel small-molecule SIRT1 inhibitors induce cell death in adult T-cell leukaemia cells. Sci. Rep..

[45] Yang Y (2017). MicroRNA-141 targets Sirt1 and inhibits autophagy to reduce HBV replication. Cell Physiol. Biochem..

