# Supplementary figures and images for: sTim-3 alleviates liver injury via regulation of the immunity microenvironment and autophagy
Source: Cell Death Discov. 2020 Jul 22;6:62. doi: 10.1038/s41420-020-00299-7 (PMC7376253; doi:10.1038/s41420-020-00299-7)

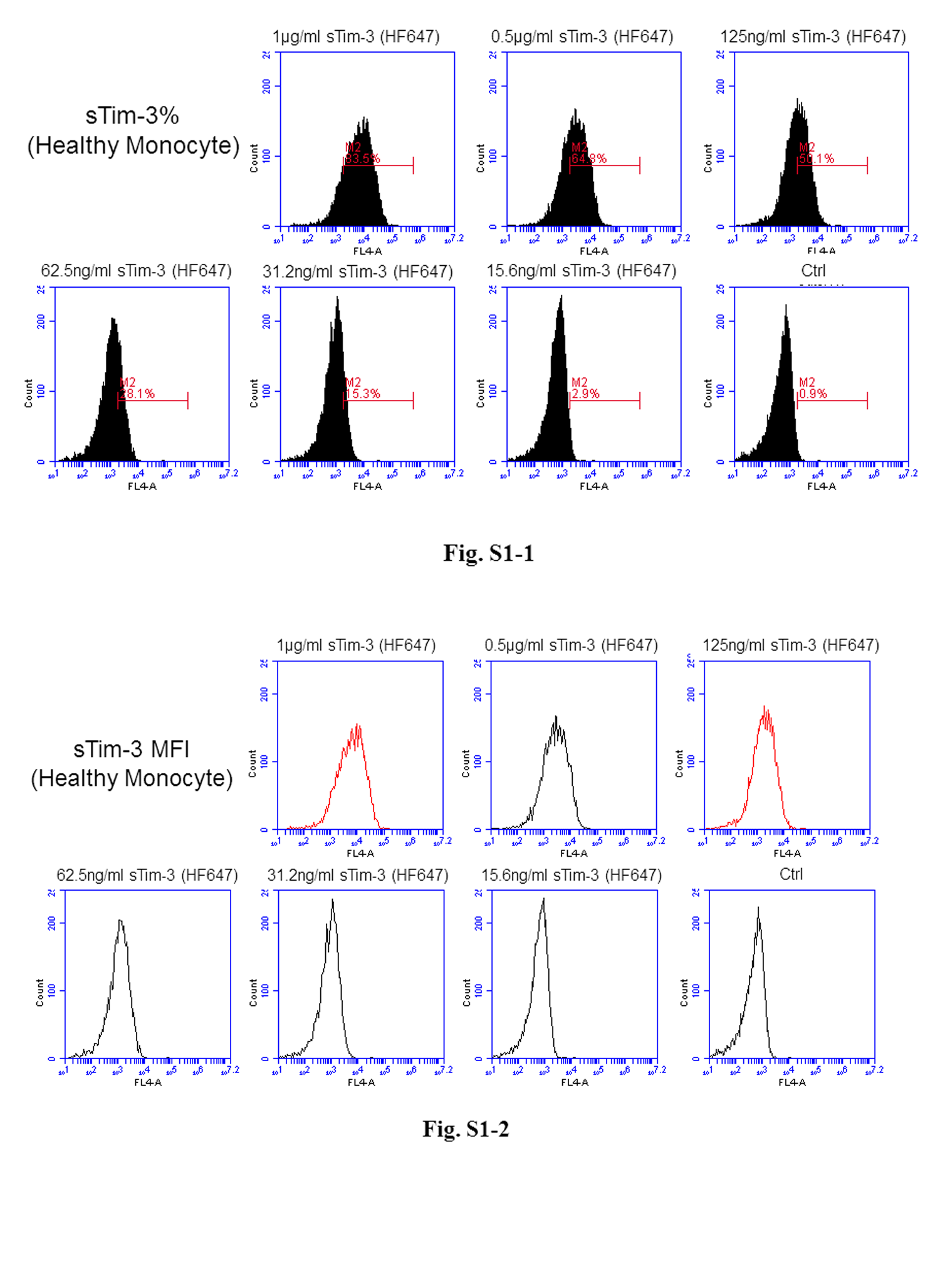

Supplement: Supplementary file 1 — Supplementary Figure 1 [file 41420_2020_299_MOESM1_ESM.tif]

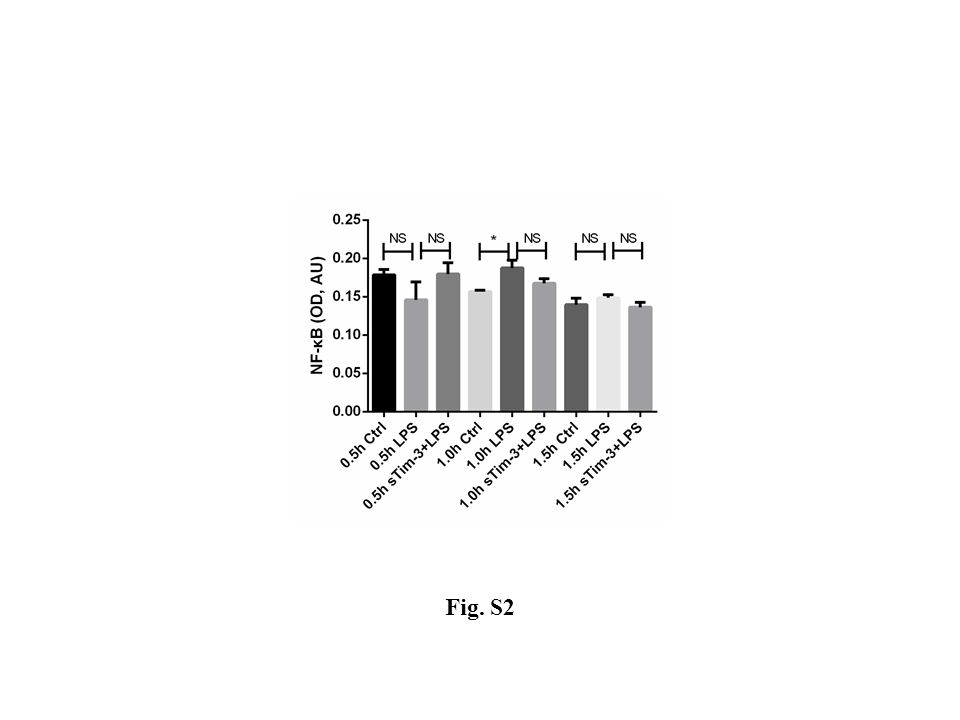

Supplement: Supplementary file 2 — Supplementary Figure 2 [file 41420_2020_299_MOESM2_ESM.tif]

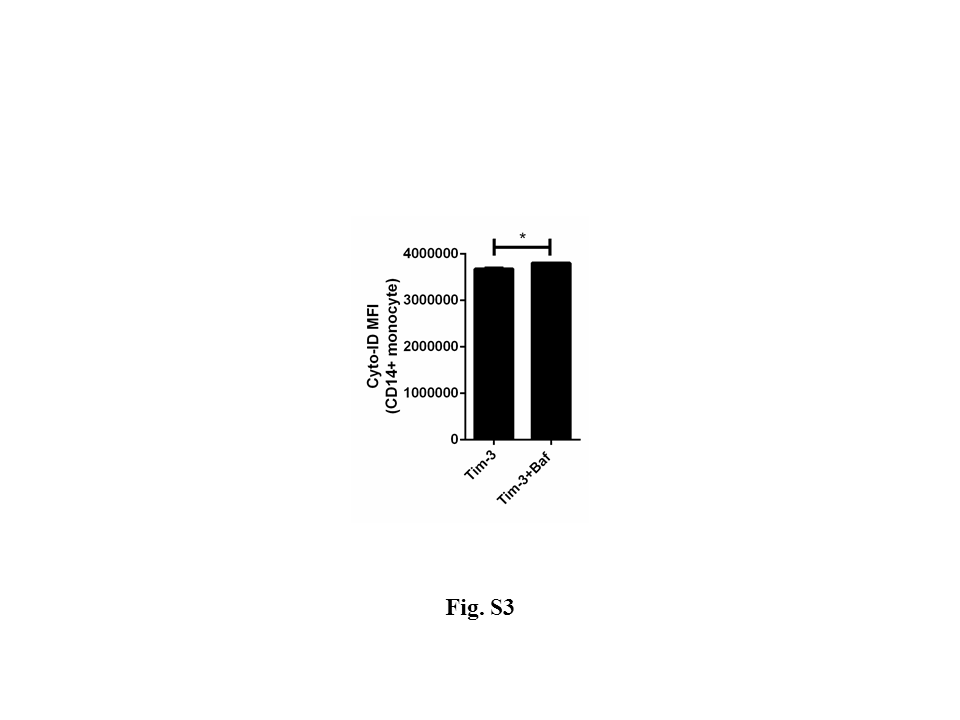

Supplement: Supplementary file 3 — Supplementary Figure 3 [file 41420_2020_299_MOESM3_ESM.tif]

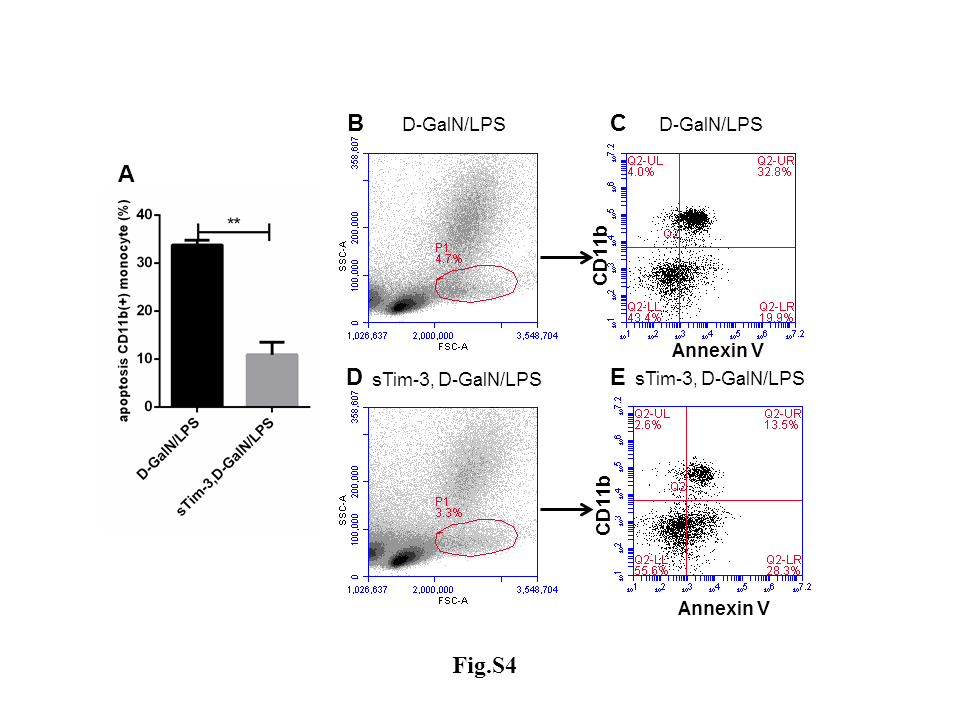

Supplement: Supplementary file 4 — Supplementary Figure 4 [file 41420_2020_299_MOESM4_ESM.tif]
