# Supplementary material for: sTim-3 alleviates liver injury via regulation of the immunity microenvironment and autophagy
Source: Cell Death Discov. 2020 Jul 22;6:62. doi: 10.1038/s41420-020-00299-7 (PMC7376253; doi:10.1038/s41420-020-00299-7)
Supplement: Supplementary file 5 — Supplementary figure legends [file 41420_2020_299_MOESM5_ESM.docx]

**Fig.S1**

**The representative flow cytometric plots of infiltrated sTim-3 into the monocytes in dose dependent manner**

**Fig.S1-1** The percentage of sTim-3 (HF647); **Fig.S1-2** The mean fluorescence intensity (MFI) of sTim-3 (HF647).

**Fig.S2**

**The phosphorylation of NF-κB in the monocytes**

The healthy CD14^+^ monocytes activated by 1μg/ml LPS were treated with 10ng/ml recombinant sTim-3 for 0.5h, 1h and 1.5h. Then the NF-κB phosphorylation of monocytes were detected by ELISA.

Data are expressed as the mean±SEM. Statistical analysis was performed by the independent t test. * *p* <0.05; NS, not statistically significant.

**Fig.S3**

**The autophagy flux with Cyto-ID MFI in the monocytes**

The healthy CD14^+^ monocytes were treated with 10ng/ml recombinant sTim-3 and 100nM Baf A1 for 6h and detected by flow cytometry using Cyto-ID dye.

Data are expressed as the mean±SEM. Statistical analysis was performed by the independent t test. * *p* <0.05.

**Fig.S4**

**The apoptosis levels in the blood monocytes influenced by recombinant sTim-3**

There were two groups: (a) D-GalN/LPS group; (b) recombinant sTim-3-treated D-GalN/LPS group. 6h after D-GalN/LPS challenge, blood were collected. The apoptosis levels of blood CD11b monocytes with Annexin V were detected by flow cytometry.

**(A)** The apoptosis percentage of CD11b monocytes. **(B-E)** Representative flow cytometric plots.

***p* <0.01. The data for statistical differences were analyzed by the independent t test.
